# Delocalisation of Majorana quasiparticles in plaquette–nanowire hybrid system

**DOI:** 10.1038/s41598-019-49227-5

**Published:** 2019-09-10

**Authors:** Aksel Kobiałka, Tadeusz Domański, Andrzej Ptok

**Affiliations:** 1 Institute of Physics, M. Curie-Skłodowska University, pl. M. Skłodowskiej-Curie 1, 20-031 Lublin, Poland; 2 Institute of Nuclear Physics, Polish Academy of Sciences, ul. W. E. Radzikowskiego 152, 31-342 Kraków, Poland

**Keywords:** Topological matter, Surfaces, interfaces and thin films, Topological matter

## Abstract

Interplay between superconductivity, spin-orbit coupling and magnetic field can lead to realisation of the topologically non–trivial states which in finite one dimensional nanowires are manifested by emergence of a pair of zero-energy Majorana bound states. On the other hand, in two dimensional systems the chiral edge states can appear. We investigate novel properties of the bound states in a system of *mixed dimensionality*, composed of one-dimensional nanowire connected with two-dimensional plaquette. We study this system, assuming either its part or the entire structure to be in topologically non–trivial superconducting state. Our results show delocalisation of the Majorana modes, upon leaking from the nanowire to the plaquette with some tendency towards its corners.

## Introduction

Recent nanotechnological progress allows for fabrication of artificial nanostructures^1^, where unique quantum phenomena and new states of matter^2^ could be observed. Prominent examples are the Majorana bound states (MBS) emerging on quasi-one-dimensional structures, e.g. semiconducting–superconducting hybrid nanowire^3–10^ or nanochains of magnetic atoms deposited on superconducting surface^11–16^. Such MBS are characterized by particle–antiparticle indistinguishability and their non–Abelian statistics^17^, which makes them promising entity for realisation of the topological quantum computing^18,19^.

Proximity-induced superconductivity combined with the magnetic field and the spin-orbit coupling (SOC) drives the system from its topologically trivial to non–trivial superconducting phase^20^. Such transition occurs at critical magnetic field *h*_*c*_, dependent on the SOC strength and dimensionality of the system^21–23^. Spectroscopically it is manifested by a coalescence of one pair of the Andreev (finite-energy) bound states into the Majorana (zero–energy) quasiparticles^24,25^.

Emergence of the degenerate Majorana modes from the Andreev bound states has been also reported in hybrid structures, comprising the quantum dots side-attached to the topological superconducting nanowires^7^. Initial theoretical prediction of such MBS *leakage* on the quantum dot region^26^ has been investigated by various groups^27–35^. In these hybrid structures the wavefunction of Majorana quasiparticle is spread onto a region of the normal quantum dot–superconducting nanowire interface^20,24,35–38^, diluting the spatial distribution of its spectral weight. This issue has been a subject of intensive experimental and theoretical studies^39^.

In one-dimensional structures the Majorana quasiparticles localise at the sample boundaries^40^ or near internal defects^32,41,42^. Contrary to that, for quasi two-dimensional systems there have been predicted chiral edge modes^43–46^ enabling the Majoranas to be delocalised, both in the real and momentum spaces^32^. Evidence for such dispersive Majorana modes have been recently provided by STM measurements for magnetic islands deposited on superconducting substrates^47–50^. Another route to achieve the topological superconductivity and MBS in two-dimensional systems relies on the phase biased planar Josephson junctions, which confine the narrow strip of electron gas subject to the Rashba interaction and magnetic field^51,52^.

**Figure 1:**
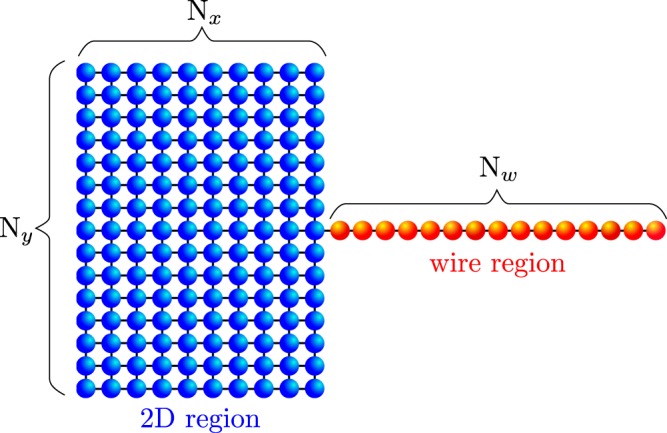
Scheme of the hybrid structure, where a semiconducting Rashba nanowire (comprising *N*_*w*_ sites) is connected to 2D cluster (whose dimensions are ${N}_{x}\times {N}_{y}$). This system is deposited on a surface of the superconducting substrate.

In general, realisation of the MBS might not be restricted solely to systems with simple geometries^46,53,54^, therefore, we propose the setup of *mixed dimensionality*, comprising one-dimensional nanowire coupled to two-dimensional plaquette (Fig. 1). This situation resembles the recently investigated nanostructures, where quasi-one-dimensional wires are attached to larger structures^8^. Here, we study the subgap spectrum of this system, focusing on spatial profiles of the Majorana modes leaking from the nanowire into the adjoined plaquette. We explore the quasiparticle spectra of this setup for representative values of the chemical potentials of both constituents (tunable by electrostatic potentials), which control topological nature of their superconducting phase. Our study gives an insight into non–local character of the Majorana quasiparticles. Proposed system can be realized experimentally in form of semiconducting–superconducting nanostrucure^8^, while results obtained in this paper can be verified experimentally.

The paper is organised as follows. First we introduce the microscopic model and present computational details. Next, we describe the numerical results obtained for each constituent and for the entire hybrid structure in various topological states. Beyond the scope of numerical calculations, we also described the proposal for experimental verification of out theoretical predictions. Finally, we summarise the results in last section.

## Model and Method

The nanostructure shown in Fig. 1 can be modelled by the real space Hamiltonian ${\mathcal H} ={ {\mathcal H} }_{kin}+{ {\mathcal H} }_{sc}+{ {\mathcal H} }_{soc}$. The first term describes the kinetic energy:

1 $${ {\mathcal H} }_{kin}=\sum _{ij\sigma }\,\{-t{\delta }_{\langle i,j\rangle }-({\mu }_{i}+\sigma h){\delta }_{ij}\}{c}_{i\sigma }^{\dagger }{c}_{j\sigma },$$

where *t* denotes the hopping integral between nearest-neighbour sites and ${c}_{i\sigma }^{\dagger }$ (*c*_*iσ*_) describes creation (annihilation) of electron on *i*-th site with spin *σ*. In general, the chemical potential *μ*_*i*_ can be tuned *in-situ* by some external gate voltage. For simplicity, however, we assume it to be constant over the entire 2D plaquette (${\forall }_{i\in 2D}\,{\mu }_{i}={\mu }_{2d}$) and in the 1D nanowire (${\forall }_{i\in w}\,{\mu }_{i}={\mu }_{w}$). We assume the Zeeman magnetic field *h* to be parallel along the wire and neglect any orbital effects^55^. The second term

2 $${ {\mathcal H} }_{sc}=\Delta \,\sum _{i}\,({c}_{i\downarrow }{c}_{i\uparrow }+{c}_{i\uparrow }^{\dagger }{c}_{i\downarrow }^{\dagger }),$$

accounts for the proximity induced on-site pairing, where Δ is the uniform energy gap in the system. The spin–orbit coupling (SOC) is given by^56–59^

3 $${ {\mathcal H} }_{soc}=\lambda \,\sum _{i\sigma \sigma ^{\prime} }\,(i{c}_{i\sigma }^{\dagger }{\sigma }_{x}^{\sigma \sigma ^{\prime} }{c}_{i+\hat{y},\sigma ^{\prime} }-i{c}_{i\sigma }^{\dagger }{\sigma }_{y}^{\sigma \sigma ^{\prime} }{c}_{i+\hat{x},\sigma ^{\prime} }+{\rm{H}}.{\rm{c}}.),$$

where *σ*_*i*_ are the Pauli matrices and *λ* stands for the Rashba potential. Since the nanowire is oriented along $\hat{x}$ axis, only the second part of SOC term survives.

Hamiltonian ${\mathcal H}$ of the hybrid structure can be diagonalised by the Bogoliubov–Valatin transformation^60^

4 $${c}_{i\sigma }=\sum _{n}\,({u}_{in\sigma }{\gamma }_{n}-\sigma {v}_{in\sigma }^{\ast }{\gamma }_{n}^{\dagger }),$$

where *γ*_*n*_, ${\gamma }_{n}^{\dagger }$ are the new *quasi*-particle fermionic operators and *u*_*inσ*_, *v*_*inσ*_ are the corresponding eigenvectors. From this transformation (4) we get the Bogoliubov–de Gennes (BdG) equations

5 $${ {\mathcal E} }_{n}(\begin{array}{l}{u}_{in\uparrow }\\ {v}_{in\downarrow }\\ {u}_{in\downarrow }\\ {v}_{in\uparrow }\end{array})=\sum _{j}\,(\begin{array}{llll}{H}_{ij\uparrow } & {D}_{ij} & {S}_{ij}^{\uparrow \downarrow } & 0\\ {D}_{ij}^{\ast } & -{H}_{ij\downarrow }^{\ast } & 0 & {S}_{ij}^{\downarrow \uparrow }\\ {S}_{ij}^{\downarrow \uparrow } & 0 & {H}_{ij\downarrow } & {D}_{ij}\\ 0 & {S}_{ij}^{\uparrow \downarrow } & {D}_{ij}^{\ast } & -{H}_{ij\uparrow }^{\ast }\end{array})(\begin{array}{l}{u}_{jn\uparrow }\\ {v}_{jn\downarrow }\\ {u}_{jn\downarrow }\\ {v}_{jn\uparrow }\end{array}),$$

where ${H}_{ij\sigma }=-\,t{\delta }_{\langle i,j\rangle }-({\mu }_{i}+\sigma h){\delta }_{ij}$ is the single-particle term, ${D}_{ij}=\Delta {\delta }_{ij}$ refers to the superconducting gap, and ${S}_{ij}^{\sigma \sigma ^{\prime} }=-\,i\lambda {({\sigma }_{y})}_{\sigma \sigma ^{\prime} }{\delta }_{\langle i,j\rangle }$ is the SOC term (which mixes particles with different spins), where ${S}_{ij}^{\downarrow \uparrow }={({S}_{ji}^{\uparrow \downarrow })}^{\ast }$ and ${S}_{ji}^{\uparrow \uparrow }={S}_{ij}^{\downarrow \downarrow }=0$.

From numerical solution of the BdG Eq. (5) we determine the Green’s function $\langle \langle {c}_{i\sigma }|{c}_{i\sigma }^{\dagger }\rangle \rangle$ and compute the local density of states (LDOS) defined as ${\rho }_{i,\sigma }(\omega )=-\,\frac{1}{\pi }\,{\rm{Im}}\,\langle \langle {c}_{i\sigma }|{c}_{i\sigma }^{\dagger }\rangle \rangle$. In the present case we have

6 $${\rho }_{i,\sigma }(\omega )=\sum _{n}\,{\xi }_{in\sigma }[\delta (\omega -{ {\mathcal E} }_{n})+\delta (\omega +{ {\mathcal E} }_{n})],$$

where the spectral weights

7 $${\xi }_{in\sigma }=|{u}_{in\sigma }{|}^{2}\theta (\,-\,{ {\mathcal E} }_{n})+|{v}_{in\sigma }{|}^{2}\theta ({ {\mathcal E} }_{n})$$

refer to probability of the *n*-th quasiparticle energy and spin *σ* to exist at *i*-th site of the system^61^.

## Numerical Results

**Figure 2:**
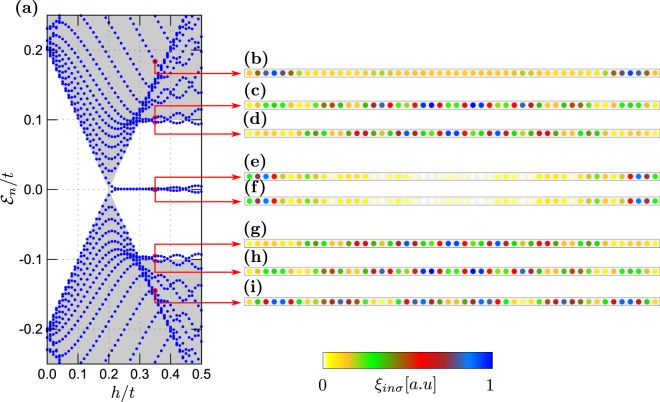
Low energy spectrum of the 1D nanowire (**a**) and the spatially resolved probabilities ${\xi }_{in}={\sum }_{\sigma }\,{\xi }_{in\sigma }$ for the quasiparticle energies ${ {\mathcal E} }_{n}$ indicated by the red arrows (**b**–**i**). Results are obtained for ${N}_{x}={N}_{y}=0$, ${N}_{w}=50$ and ${\mu }_{w}=-\,2.0t$.

**Figure 3:**
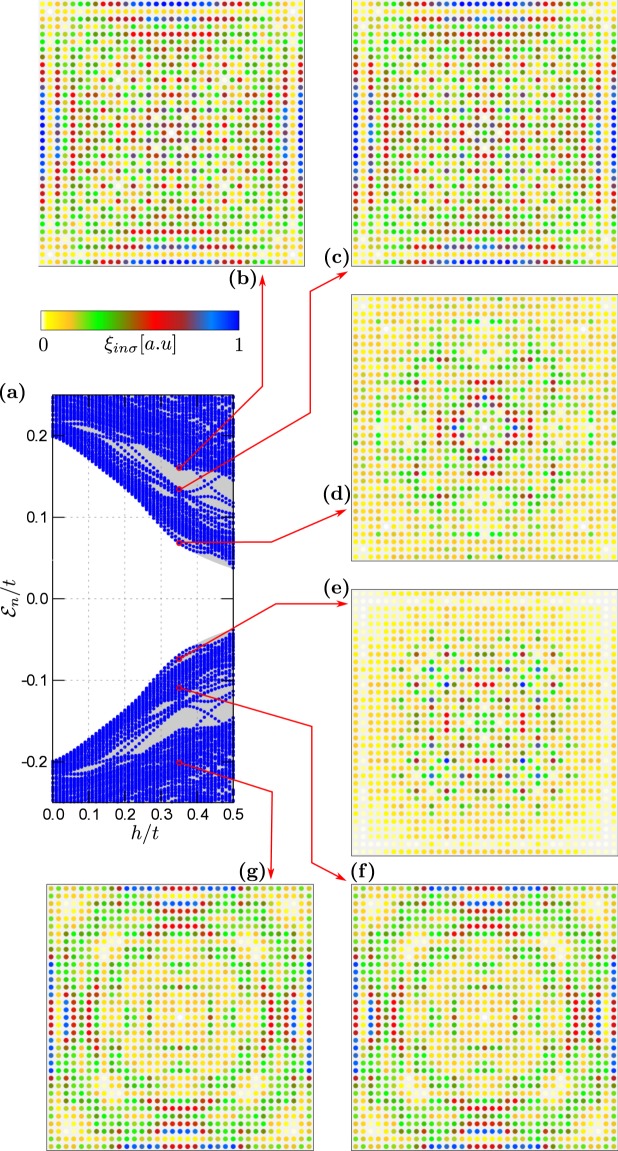
Low energy spectrum of the 2D plaquette obtained in topologically trivial state for ${\forall }_{i}{\mu }_{i}=-\,2.0t$ (**a**). Panels (b–g) display spatial profiles of the quasiparticle for several eigenvalues ${ {\mathcal E} }_{n}$. Results are obtained for $h/t=0.35$, assuming ${N}_{x}={N}_{y}=35$ and ${N}_{w}=0$.

**Figure 4:**
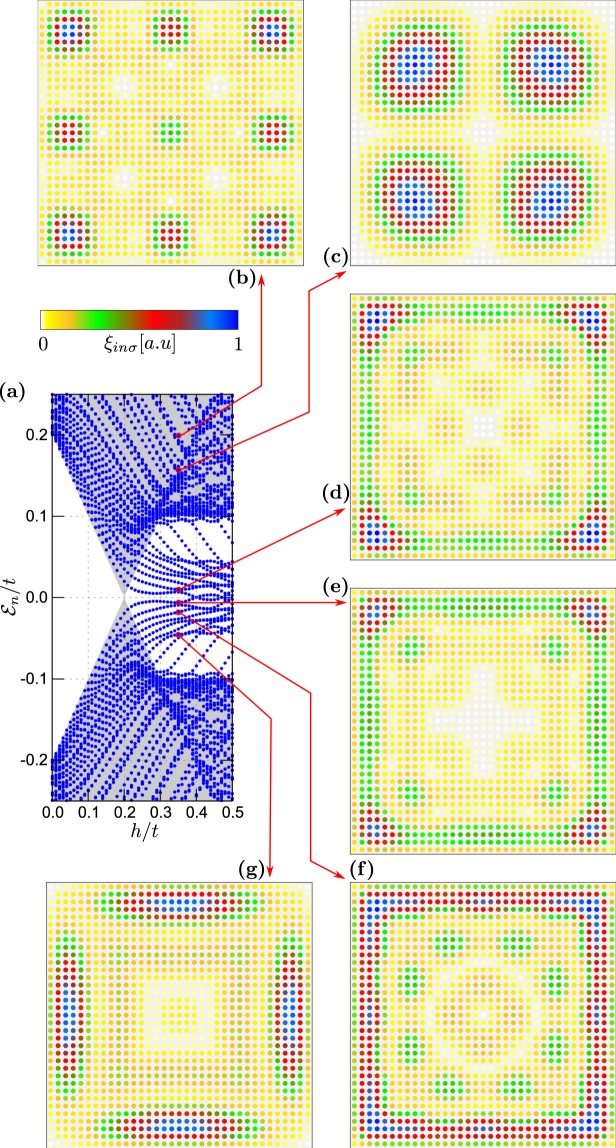
Low energy spectrum of the 2D plaquette obtained in topologically non--trivial state for ${\forall }_{i}{\mu }_{i}=-\,4.0t$ (**a**). All other model parameters are the same as in Fig. 3.

**Figure 5:**
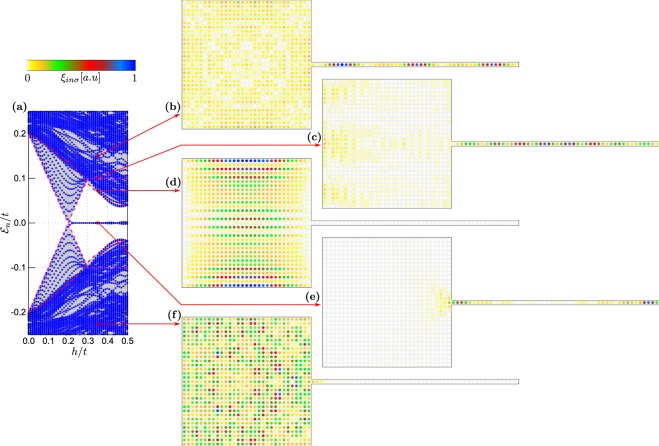
Spectrum of the plaquette–nanowire hybrid system obtained for ${N}_{x}={N}_{y}=31$ and ${N}_{w}=50$, $h=0.35t$, assuming ${\mu }_{2D}={\mu }_{w}=-\,2.0t$. In this case, only the nanowire is in the non--trivial topological superconducting phase.

**Figure 6:**
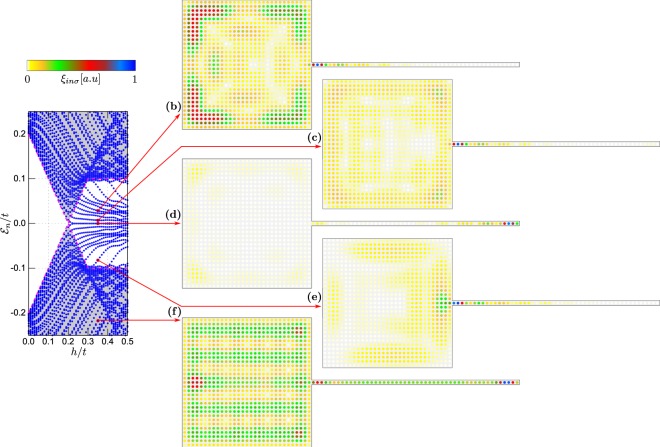
Spectrum of the plaquette–nanowire hybrid system obtained for ${N}_{x}={N}_{y}=31$ and ${N}_{w}=50$, $h=0.35t$, assuming ${\mu }_{2D}=-\,4.0t$ and ${\mu }_{w}=-\,2.0t$, which guarantee that both constituents are in the non–trivial topological phase.

In what follows, we study the hybrid setup consisting of $N={N}_{x}\times {N}_{y}+{N}_{w}$ sites, which is ${N}_{x}\times {N}_{y}$ sites of the 2D–plaquette and *N*_*w*_ sites in of 1D–nanowire. For numerical computations we choose Δ/*t* = 0.2 and *λ*/*t* = 0.15. In presence of the spin-orbit coupling and the Zeeman effect the on-site electron pairing evolves into the inter-site (*p*–wave) superconducting phase^23,62–64^. Its topological form occurs above the critical magnetic field ${h}_{c}=\sqrt{{\Delta }^{2}+{(-W\pm \mu )}^{2}}$^21,22^, where *W* is half of the bandwidth (equal to 2*t* and 4*t* for 1D and 2D system, respectively). Upon increasing the magnetic field *h*, the superconducting gap closes at *h*_*c*_ and reopens when entering the topological region. Our calculations are done for the finite-size system, therefore the quasiparticle spectra are discretised. As a useful guide-to-eye, in panels (a) of Figs 2–6 we have marked the continuous spectrum of the bulk system by grey colour.

Described system can be experimentally implemented in the form of the semiconducting–superconducting hybrid nanostructure. In such heterostructure the realistic parameters can be estimated as^4,5,65^: superconducting gap Δ $\simeq$ 250 *μ*eV, SOC strength *λ* $\simeq$ 0.25 eV · Å, and effective mass of electrons m* ≈ 0.15 *m*_*e*_. Topological phase transition in the nanowire is observed for *h* ~ 0.15 t, when magnetic field starts to exceed Δ. Independently from realistic values of physical quantities, parameter values assumed in our calculations allow for clear exhibition of behavior and physical properties of described system.

### Separate components of the system

Here, we briefly describe the quasiparticles for each component of the hybrid structure separately. Let us start with the 1D chain. For ${\forall }_{i}{\mu }_{w}=-\,2.0t$ and assuming the magnetic field $h=0.35t$ the nanowire would be in its non–trivial topological state. Figure 2(b)–(i) show the spatial distribution for representative quasiparticle states whose energies are indicted by the arrows. The quasiparticles from outside the topological gap [panels (b)–(d) and (g)–(i)] are spread all over the nanowire, whereas two states residing inside the topological gap [panels (e) and (f) in Fig. 2] are clearly localised near the nanowire ends. Such zero-energy quasiparticles exist only in the topological region, above the critical magnetic field ${h}_{c}=0.2t$, and can be identified as the MBS. Let us notice, that quasiparticles at energies $\pm { {\mathcal E} }_{n}$ have the same spatial patterns [cf. panels (e) and (f), (d) and (g), or (c) and (h)], due to electron–hole symmetry of the BdG equations.

Let’s now focus on properties of the plaquette. Figure 3 shows the spectrum and displays the profiles of selected quasiparticles obtained for ${\forall }_{i}{\mu }_{i}=-\,2.0t$ when the 2D-region is in a trivial superconducting phase. Under such circumstances, there is no evidence for any in--gap quasiparticles regardless of *h*. Figure 4 presents the results obtained ${\forall }_{i}{\mu }_{i}=-\,4.0t$, corresponding to the non--trivial superconducting phase. By inspecting the quasiparticles outside the topological gap [panels (b)–(c)] we observe their nearly uniform distribution in the plaquette without clear signatures of any edge phenomena. On the other hand, the quasiparticle states existing inside the topological gap [panels (d)–(g)] reveal a tendency towards their localisation near the sample boundaries^43–46^.

It is worth noting, that the in both cases, the transition from trivial to topological phase is associated with closing of the soft-superconducting gap at some critical magnetic field *h*_*c*_^21–23^. For chosen parameters, in the 1D-nanowire (Fig. 2a) and 2D-plaquette (Fig. 4a) these fields are equal and given as *h*_*c*_/*t* $\simeq$ 0.2. For magnetic field $h > {h}_{c}$ new topological gap is reopened. This allows for simultaneous emergence of in--gap states in the both constituents of finite system (with edges).

Appearance of the MBS in the nanowire can be associated with changes of topological ${{\mathbb{Z}}}_{2}$ index^66^. Furthermore, we know that the MBS must always appear in pairs in the nanowires. Contrary to this, in the 2D-plaquette we observe plenty of in–gap states whose energies differ from zero. Strictly speaking, we do not observe the completely localised Majorana states in such 2D systems. This situation changes qualitatively, however, in the plaquette–nanowire hybrid system.

### Plaquette-nanowire hybrid

Let us first consider the case, when only part of our hybrid setup is in the topologically non–trivial superconducting phase. This can be realised e.g. for the chemical potential ${\mu }_{2D}={\mu }_{w}=-\,2t$ (Fig. 5). For the chosen model parameters, the critical magnetic field of the nanowire is ${h}_{c}=0.2t$. In this case the quasiparticle spectrum shows a collection of levels, originating from the 1D and 2D regions [cf. with Figs 2 and 4]. By increasing the magnetic field the original gap closes at $h={h}_{c}$ and at stronger magnetic fields the nanowire part is in the non–trivial topological phase. In consequence, we observe emergence of just one pair of the nearly–zero–energy bound states originating from the 1D part of our setup [Fig. 5(e)]. One of these quasiparticles is localised at interface with the nanowire region and partly leaks into the 2D plaquette. Other (finite-energy) quasiparticles are distributed either over the plaquette [Fig. 5(d)] or over both regions of the setup [Fig. 5(b,c)].

Below, we describe the results for the system, in which both 1D and 2D regions simultaneously undergo a transition to non–trivial topological phase. This situation can be achieved by fine tuning the site dependent chemical potentials – choosing ${\mu }_{w}=-\,2t$ and ${\mu }_{2D}=-\,4t$ yields a transition to topological phase for the entire system at ${h}_{c}=0.2t$. Numerical results for this case are shown in Fig. 6. By inspecting the lowest in--gap states for $h > {h}_{c}$ we observe their localisation near the boundaries of the system, i.e. at a free-standing end of the nanowire (right-hand side of the system) and in corners of the plaquette. On the other hand, the states from the electron band regions are nearly uniformly spread over the whole structure [Fig. 6(f)].

It is worth noting that both parts of the system have comparable topological gap. Due to existence of the common topological non--trivial state, all the in--gap states tend to be localised at the sample edges. The quasiparticle state appearing at zero energy is predominantly localised in the right hand end of the wire [Fig. 6(d)], whereas its co-partner (initially localised at the left side of nanowire) partly *leaks* onto the adjoined 2D-region and appears predominantly in the corners of the plaquette. Contrary to the previous case [displayed in Fig. 5(e)] the MBS are strongly delocalised and redistributed. The other finite–energy states appear either near the wire-plaquette boundary [see Fig. 6(c,e)] or at the edges of the plaquette.

#### Role of finite size effects

**Figure 7:**
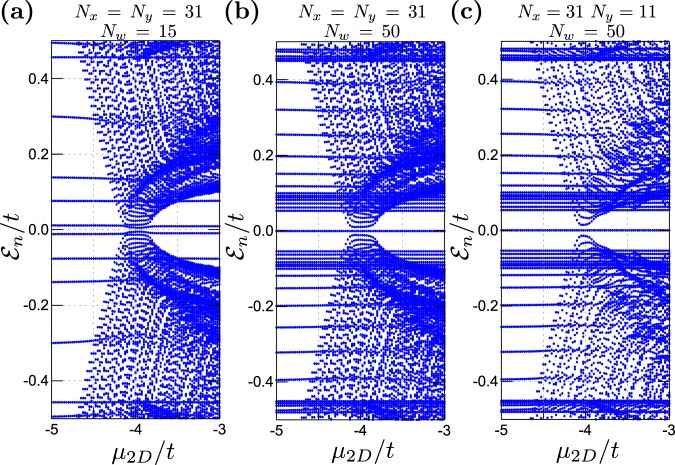
Spectrum of the plaquette–nanowire hybrid as a function of *μ*_2*D*_ for various sizes of 1D and 2D components (as indicated) obtained for $h/t=0.25$ and ${\mu }_{w}/t=-\,2$.

Here, we address influence of the finite-size of our hybrid structure. In Fig. 7 we show the eigenvalues for three different sizes of the system as a function of the chemical potential *μ*_2*D*_. Let us remark, that plaquette is in the non–trivial topological phase for ${\mu }_{2D}/t-4.0$. Emergence of the in--gap states is well visible in all cases as can be seen by the horizontal zero-energy lines that correspond to the quasiparticles originating from the nanowire (for which we have fixed the chemical potential at ${\mu }_{w}/t=-\,2$). If a nanowire is very short, the MBS overlap with each other, forming the bonding and anti-bonding states [Fig. 7(a)]. Consequence of such overlapping wavefunctions have been studied by a number of authors^67–69^. In some analogy to this behaviour, also variation of the plaquette size ${N}_{x}\times {N}_{y}$ can lead to rearrangement of the quasiparticle states, depending on *μ*_2*D*_. In particular, it may reduce a number of the in–gap states which appear near the sample edges.

#### Nanowire coupled to plaquette’s corner

**Figure 8:**
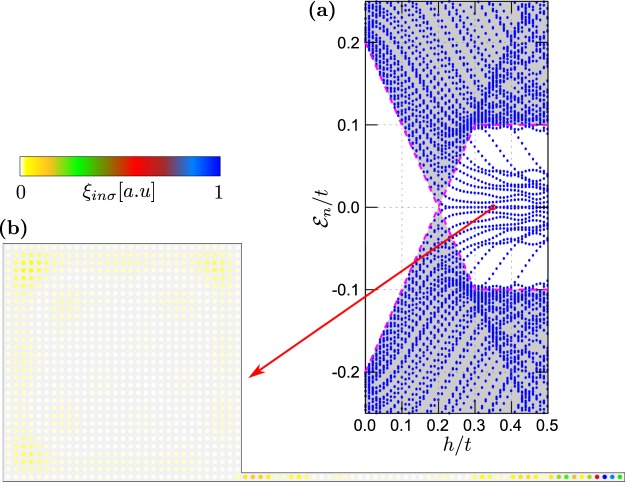
Spectrum of the plaquette–nanowire hybrid obtained for ${N}_{x}={N}_{y}=31$, ${N}_{w}=50$, in the case when the nanowire is connected to the corner of the 2D-region.

We have also checked numerically, that spatial patterns of the MBS leaking from 1D to 2D parts do not depend strongly on a particular location of the contact point between these constituents. In Fig. 8 we illustrate this effect, considering the hybrid structure where the nanowire is attached to a corner of the plaquette. In this situation the delocalised MBS accumulates near three other corners of the plaquette, yet some of its remnants are still observable at the interface with nanowire. Irrespectively of the particular contact point, MBS is again strongly delocalised [cf. Fig. 6(d)].

## Proposal for Empirical Detection

Quasiparticles of the topologically non–trivial superconducting state appearing at zero energy at boundaries of one and two-dimensional parts in our hybrid structure could be experimentally probed by the scanning tunnelling microscopy (STM). Its low-energy and spin-polarised version, relying on the *selective equal–spin Andreev reflection* (SESAR), has been proposed^70^ as a unique tool for probing the Majorana quasiparticles manifested by the zero-bias tunnelling conductance. This kind of STM measurements, using ferromagnetic tip, has been already sucessfully used in the study of the Majorana bound states in the monoatomic ferromagnetic chain deposited at the superconducting surface^15^.

Let us briefly explain how such SESAR spectroscopy could probe the spatial distribution of the localised and delocalised Majorana quasiparticles in our hybrid structure. Applying voltage *V* between the conducting STM tip and the superconducting nanowire-plaquette system induces the charge transport of a given spin *σ* carriers. On microscopic level, electrons arriving from the STM tip would be converted into the inter-site pairs, reflecting holes of the same spin polarisation back to the tip. The resulting current can be expressed by the Landauer–Büttiker formula^71^:

8 $${I}_{i,i+1}^{\sigma }(V)=\frac{e}{h}\,{\int }^{}\,{T}_{i,i+1}^{\sigma }(\omega )[f(\omega -eV)-f(\omega +eV)]d\omega ,$$

where the transmittance for a given pair of the neighbouring sites is ${T}_{i,i+1}^{\sigma }(\omega )={|{\Gamma }_{N}^{\sigma }|}^{2}\,{|\langle \langle {\hat{d}}_{i\sigma };{\hat{d}}_{i+1\sigma }\rangle \rangle |}^{2}$ with ${\Gamma }_{N}^{\sigma }$ denoting the spin-dependent hybridisation between the STM tip and individual sites of our hybrid structure. We assume it to be uniform, which should be reasonable assumption as long as distance between the STM tip and the probed system is kept constant.

At low temperatures the conductance simplifies to

9 $${G}_{i,i+1}^{\sigma }(V)\equiv \frac{d}{dV}{I}_{i,i+1}^{\sigma }(V)\simeq \frac{2{e}^{2}}{h}{T}_{i,i+1}^{\sigma }(\omega =eV).$$

**Figure 9:**
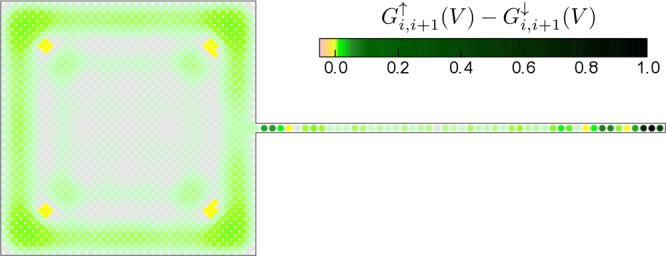
Difference of the zero-bias tunnelling conductance between ↑ and ↓ charge carriers obtained in units of 2*e*^2^/*h*, assuming both parts of our hybrid system to be in topologically non–trivial superconducting phase.

Figure 9 shows a difference of the spin polarised conductance ${G}_{i,i+1}^{\uparrow }(V)-{G}_{i,i+1}^{\downarrow }(V)$ obtained at zero bias $V=0$. Transmittance ${T}_{i,i+1}^{\sigma }(\omega )$ of the SESAR has been computed for all pairs of the neighboring sites, both in the nanowire and in the nanoscopic plaquette using the off-diagonal Green’s function determined from the BdG diagonalization procedure. Each point presented at Fig. 9 corresponds to the central place between the neighboring *i* and *i* + 1 sites, both in *x* and *y* directions. We clearly see that this polarised zero-bias conductance is strongly enhanced near the localised Majorana mode at the end of nanowire and can also allow for detection of its delocalised partner, whose spectral weight is smeared along the boundaries of 2D-plaquette. Additionally, it should be noted that the magnitude of spin polarised conductance is locally 100 times lower in plaquette than in nanowire.

Other proposal for probing the delocalised mode could be also based on measurements of the edge currents^44,72,73^, however we can hardly judge its practical feasability. Such local supercurrents between the topologically trivial and non–trivial parts of the proximitized systems have been also addressed in ref.^74^ and several important aspects concerning the topological superconductivity of 2D systems have been discussed in refs^75^ and^76^.

## Summary

We have investigated quasiparticle spectra of the hybrid system, comprising the 1D-nanowire attached to the 2D-plaquette, both proximitized to the s-wave superconductor. Depending on the electron energies in these constituents (which should be tunable by external gate potentials), the spin-orbit interactions along with the magnetic field could induce the topologically non–trivial superconducting phase, either (i) only in the nanowire or (ii) in the entire setup. Selfconsistent numerical determination of the quasiparticle spectra has revealed, that under such circumstances the zero-energy Majorana quasiparticles would be (i) localised near the ends of 1D-nanowire or (ii) one of them would leak into the plaquette region. For the latter case we have inspected the spatial profile of the delocalised Majorana mode and found its signatures distributed along boundaries of the 2D-plaquette, with some preference towards its corners. We have shown that, spatial profiles of both the localised and delocalised Majorana quasiparticles could be probed by the polarised scanning tunnelling measurements, where such quasiparticles would be detectable via the Andreev scattering mechanism.

Proposed hybrid structure (and similar ones) could open new perspectives for studying the topological superconducting phase in complex geometries of mixed dimensionality and might shed an insight into itinerancy of the emerging Majorana modes^46,77^. Furthermore, such delocalisation of the Majorana modes could be practically used for realisation of their braiding by attaching a few nanowires to the plaquette region.

### Additional note

Our proposal refers to the semiconductor–superconductor hybrid nanostructure. However, after the initial version of this manuscript has been submitted, similar concept have been proposed in context of the recent experiments concerning the magnetic nanoflakes^77^. This proposal is strongly associated with realization of the zero energy bound state around two dimensional magnetic structure^47,49^.
